# Stochastic Order and Generalized Weighted Mean Invariance

**DOI:** 10.3390/e23060662

**Published:** 2021-05-25

**Authors:** Mateu Sbert, Jordi Poch, Shuning Chen, Víctor Elvira

**Affiliations:** 1 Graphics and Imaging Laboratory, University of Girona, 17003 Girona, Spain; jordi.poch@udg.edu; 2 Department of Civil Engineering, Urban Institute, School of Engineering, Kyushu University, Fukuoka 819-0395, Japan; njushuningchen@hotmail.com; 3 School of Mathematics, University of Edinburgh, Edinburgh EH9 3FD, UK; victor.elvira@ed.ac.uk

**Keywords:** stochastic order, weighted mean, arithmetic mean, harmonic mean, Kolmogorov mean, quasi-arithmetic mean, cross-entropy, Shannon entropy, Rényi entropy, Tsallis entropy, multiple importance sampling

## Abstract

In this paper, we present order invariance theoretical results for weighted quasi-arithmetic means of a monotonic series of numbers. The quasi-arithmetic mean, or Kolmogorov–Nagumo mean, generalizes the classical mean and appears in many disciplines, from information theory to physics, from economics to traffic flow. Stochastic orders are defined on weights (or equivalently, discrete probability distributions). They were introduced to study risk in economics and decision theory, and recently have found utility in Monte Carlo techniques and in image processing. We show in this paper that, if two distributions of weights are ordered under first stochastic order, then for any monotonic series of numbers their weighted quasi-arithmetic means share the same order. This means for instance that arithmetic and harmonic mean for two different distributions of weights always have to be aligned if the weights are stochastically ordered, this is, either both means increase or both decrease. We explore the invariance properties when convex (concave) functions define both the quasi-arithmetic mean and the series of numbers, we show its relationship with increasing concave order and increasing convex order, and we observe the important role played by a new defined mirror property of stochastic orders. We also give some applications to entropy and cross-entropy and present an example of multiple importance sampling Monte Carlo technique that illustrates the usefulness and transversality of our approach. Invariance theorems are useful when a system is represented by a set of quasi-arithmetic means and we want to change the distribution of weights so that all means evolve in the same direction.

## 1. Introduction and Motivation

Stochastic orders [1,2], are orders defined in probability theory and statistics, to quantify the concept of one random variable being *bigger* or *smaller* than another one. Discrete probability distributions, also called probability mass functions (pmf), are sequences of n-tuples of non-negative values that add up to 1, and can be thus interpreted in several ways, for instance, as weights in computation of moments of the discrete random variable described by the pmf, or as equivalence classes of compositional data [3]. Stochastic orders have found application in decision and risk theory [4], and in economics in general, among many other fields [2]. Some stochastic orders have been defined based on order invariance: two pmf’s are ordered when the arithmetic means of any increasing sequence of real numbers weighted with the corresponding pmf’s are ordered in the same direction. This raises the question whether this invariance might also hold for other kind of means beyond the arithmetic mean.

The quasi-arithmetic means, also called Kolmogorov or Kolmogorov–Nagumo means, are ubiquitous in many branches of science [5]. They have the expression $g^{-1}\left(\sum_{k=1}^{M}\alpha_{k}g\left(b_{k}\right)\right)$, where g(x) is a real-valued strictly monotonous function, ${\left\{b_{k}\right\}}_{k=1}^{M}$ a sequence of reals, and ${\left\{\alpha_{k}\right\}}_{k=1}^{M}$ a set of weights with $\sum_{k=1}^{M}\alpha_{k}=1$. This family of means comprises the usual means: arithmetic g(x)=x, $\sum_{k=1}^{M}\alpha_{k}b_{k}$, harmonic g(x)=1/x, ${\left(\sum_{k=1}^{M}\frac{\alpha_{k}}{b_{k}}\right)}^{-1}$, power mean $g\left(x\right)=x^{p}$, ${\left(\sum_{k=1}^{M}\alpha_{k}b_{k}^{p}\right)}^{\frac{1}{p}}$. For a long time, economists have discussed the best mean for a problem [6]. Harmonic mean is used for the price earning ratio, and power means are used to represent the aggregate labor demand and its corresponding wage [7], and the constant elasticity of substitution (CES) [8]. Yoshida [9,10] has studied the invariance under quasi-arithmetic means with function g(x) increasing and for utility functions. In information theory, Alfred Rényi [11] defined axiomatically the entropy of a probability distribution as a Kolmogorov mean of the information $1/\log p_{k}$ conveyed by result *k* with probability $p_{k}$, and recently, Americo et al. [12] defined conditional entropy based on quasi-arithmetic means. In physics, the equivalent spring constant of springs combined in series is obtained as the harmonic mean of the individual spring constants, and in parallel, as their arithmetic mean [13], while the equivalent resistance of resistors combined in parallel is obtained by the harmonic mean of the individual resistances, and in series by their arithmetic mean [14]. In traffic flow [15], arithmetic and harmonic mean of speed distribution are used. In [16] both geometric and harmonic mean are used in addition to arithmetic mean to improve noise source maps.

In our recent work on inequalities for generalized, quasi-arithmetic weighted means [17], we found some invariance properties, that depended on the particular relationship considered between the sequences of weights. These relationships between weights define first stochastic order, and likelihood ratio order. Their application to multiple importance sampling (MIS), a Monte Carlo technique, has been presented in [18], its application to cross entropy in [19], and in [20] applications to image processing, traffic flow and income distribution have been shown. In [21], the invariance results on products of distributions of independent scalar r.v.’s [22] was generalized to any joint distribution of a 2-dimensional r.v.

In this paper, we show that the order invariance is a necessary and sufficient condition for first stochastic order, and that it holds under any quasi-arithmetic mean. We also study invariance under the second stochastic order, likelihood ratio, hazard-rate, and increasing convex stochastic orders. The fact that the invariance results hold for both increasing and decreasing monotonic functions allows us to use both utilities and liabilities, represented by efficiencies and expected error, respectively, to look for an optimal solution, where in liabilities we look for minimum expected error, while in utilities for maximum efficiency.

The rest of the paper is organized as follows. In Section 2, we introduce the stochastic order; in Section 3, the arithmetic mean and its relationship with stochastic order. In Section 4, we present the invariance theorems; in Section 5, we discuss the invariance for concave (convex) functions; in Section 6, its application to stochastic orders; in Section 7, we present an example based on the linear combination of Monte Carlo estimators. Finally, conclusions and future work are given in Section 8.

## 2. Stochastic Orders

Stochastic orders are pre-orders (i.e, binary relationships holding symmetric and transitive properties) defined on probability distributions with finite support. Note that equivalently, one can think of sequences (i.e., ordered sets) of non-negative weights/values that sum up to one. Observe that any sequence $\left\{\alpha_{k}\right\}$ of *M* positive numbers such that $\sum_{k=1}^{M}\alpha_{k}=1$ can be considered a probability distribution. It can be seen too as an element of the (M-1)-Simplex, i.e., $\left\{\alpha_{k}\right\}\in \Delta_{M-1}$. While several interpretations hold and hence increase the range of applicability, in the remaining of this paper, we will talk of sequence without any loss of generality.

**Notation.**  *We use the symbols ≺,≻ to represent orders between two sequences $\left\{\alpha_{k}\right\}$ and $\left\{\alpha_{k}^{\prime }\right\}$ of size M, e.g., and we write $\left\{\alpha_{k}\right\}≻\left\{\alpha_{k}^{\prime }\right\}$ or equivalently $\left\{\alpha_{k}^{\prime }\right\}≺\left\{\alpha_{k}\right\}$. We will denote the elements of the sequences without the curly brackets, e.g., the first element of the sequence $\left\{\alpha_{k}\right\}$ is denoted as $\alpha_{1}$, while the last one is $\alpha_{M}$. Moreover, the first and last elements of the sequence receive special attention, since when $\alpha_{1}\leq \alpha_{1}^{\prime }$, we can write this order as $\left\{\alpha_{k}\right\}{≻}_{F}\left\{\alpha_{k}^{\prime }\right\}$ (where ${≻}_{F}$ stands for* first *) and whenever $\alpha_{M}\geq \alpha_{M}^{\prime }$, we can write $\left\{\alpha_{k}\right\}{≻}_{L}\left\{\alpha_{k}^{\prime }\right\}$ (where ${≻}_{L}$ stands for* last *). The case with both $\alpha_{1}\leq \alpha_{1}^{\prime }$ and $\alpha_{M}\geq \alpha_{M}^{\prime }$, we can denote as $\left\{\alpha_{k}\right\}{≻}_{FL}\left\{\alpha_{k}^{\prime }\right\}$. The orders ${≻}_{F},{≻}_{L},{≻}_{FL}$ are superorders of first stochastic dominance order, $\left\{\alpha_{k}\right\}{≻}_{FSD}\left\{\alpha_{k}^{\prime }\right\}$, that will be studied in Section 6. We denote as $\left\{\alpha_{M-k+1}\right\}$ the sequence with the same elements of $\left\{\alpha_{k}\right\}$ but in reversed order.*

**Example** **1** (Toy example)**.** *Given sequences $\left\{\alpha_{k}\right\}=\left\{0.5,0.1,0.2,0.2\right\}$, and $\{\alpha_{k}^{\prime }\}=${0.4,0.1,0.2,0.3}, we have both $\left\{\alpha_{k}\right\}{≺}_{F}\left\{\alpha_{k}^{\prime }\right\}$, $\left\{\alpha_{k}\right\}{≺}_{L}\left\{\alpha_{k}^{\prime }\right\}$, and thus $\left\{\alpha_{k}\right\}{≺}_{FL}\left\{\alpha_{k}^{\prime }\right\}$. On the other hand for sequences $\left\{\alpha_{k}\right\}$ and $\left\{\alpha_{k}^{\prime \prime }\right\}=\left\{0.4,0.3,0.2,0.1\right\}$, we have $\left\{\alpha_{k}\right\}{≺}_{F}\left\{\alpha_{k}^{\prime \prime }\right\}$, $\left\{\alpha_{k}\right\}{⊀}_{L}\left\{\alpha_{k}^{\prime \prime }\right\}$, and thus $\left\{\alpha_{k}\right\}{⊀}_{FL}\left\{\alpha_{k}^{\prime \prime }\right\}$.*

**Property** **1** (Mirror property)**.** *One desirable property of stochastic orders is such that when reversing the ordering of the sequence elements, the stochastic order should be reversed as well, i.e., if $\left\{\alpha_{k}\right\}≻\left\{\alpha_{k}^{\prime }\right\}$, then $\left\{\alpha_{M-k+1}^{\prime }\right\}≻\left\{\alpha_{M-k+1}\right\}$. This is similar to the invariance of physical laws to the right and left hand. We call this property the* mirror *property.*

**Definition** **1** (Mirror property)**.** *We say that a stochastic order has the mirror property if*

(1) $$\left\{\alpha_{k}^{\prime }\right\}≺\left\{\alpha_{k}\right\}\Rightarrow \left\{\alpha_{M-k+1}\right\}≺\left\{\alpha_{M-k+1}^{\prime }\right\}.$$

Observe that the simple orders defined before, ${≻}_{F}$, ${≻}_{L}$, do not hold this property, but ${≻}_{FL}$ holds it. We will see in Section 6 that usual stochastic orders do hold the mirror property. However, an order that is insensitive to the permutation of the elements of a sequence, like majorization or Lorentz order, does not hold the mirror property.

## 3. Quasi-Arithmetic Mean

Stochastic orders are usually defined by invariance to arithmetic mean [1,2] (see Section 6), and we want to investigate in this paper invariance to more general means. We define here the kind of means we are interested in.

**Definition** **2** (Quasi-arithmetic or Kolmogorov or Kolmogorov–Nagumo mean)**.** *A quasi-arithmetic weighted mean (or Kolmogorov mean) $M(\left\{b_{k}\right\},\left\{\alpha_{k}\right\})$ of a sequence of real numbers ${\left\{b_{k}\right\}}_{k=1}^{M}$, is of the form $g^{-1}\left(\sum_{k=1}^{M}\alpha_{k}g\left(b_{k}\right))\right)$, where g(x) is a real valued, invertible strictly monotonic function, with inverse function $g^{-1}\left(x\right)$ and ${\left\{\alpha_{k}\right\}}_{k=1}^{M}$ are positive weights such that $\sum_{k=1}^{M}\alpha_{k}=1$.*

Examples of such mean are arithmetic weighted mean (g(x)=x), harmonic weighted mean (g(x)=1/x), geometric weighted mean (g(x)=logx) and in general, the weighted power means ($g\left(x\right)=x^{r}$).

Given a distribution $\left\{p_{k}\right\}$, Shannon entropy, $H\left(\left\{p_{k}\right\}\right)=-\sum_{k}p_{k}\log p_{k}$, and Rényi entropy, $R_{\beta }\left(\left\{p_{k}\right\}\right)=\frac{1}{1-\beta }\log\sum_{k}p_{k}^{\beta }$, can be considered as quasi-arithmetic means of the sequence $\left\{\frac{1}{\log p_{k}}\right\}$ with weights $\left\{p_{k}\right\}$: Shannon entropy with g(x)=x (arithmetic mean or expected value), and Rényi entropy with $g\left(x\right)=2^{(\beta -1)x}$ [11]. Tsallis entropy, $S_{q}\left(\left\{p_{k}\right\}\right)=\frac{1-\sum_{i=1}^{M}p_{i}^{q}}{q-1}$, can be considered the weighted arithmetic mean of the sequence $\left\{\frac{1}{\ln_{q}p_{k}}\right\}$ with weights $\left\{p_{k}\right\}$, where $\ln_{q}x=\frac{x^{1-q}-1}{1-q}$ is the *q*-logarithm function [23]. Without loss of generality, we consider from now on that $\left\{b_{k}=f\left(k\right)\right\}$ where f(x) is a real valued function.

**Lemma** **1.** *Consider the sequences of M positive weights ${\left\{\alpha_{k}\right\}}_{k=1}^{M}$ and ${\left\{\alpha_{k}^{\prime }\right\}}_{k=1}^{M}$, $\sum_{k=1}^{M}\alpha_{k}=1,\sum_{k=1}^{M}\alpha_{k}^{\prime }=1$, g(x) a strictly monotonic function. The following conditions (a), (b), (a’), (b’), (c) and (d) are equivalent.*

*(a) for g(x) increasing, for any increasing function f(x) the following inequality holds:*

(2) $$\sum_{k=1}^{M}\alpha_{k}^{\prime }g\left(f\left(k\right)\right)\leq \sum_{k=1}^{M}\alpha_{k}g\left(f\left(k\right)\right)$$

*(b) for g(x) increasing, for any increasing function f(x) the following inequality holds:*

(3) $$\sum_{k=1}^{M}\alpha_{M-k+1}g\left(f\left(k\right)\right)\leq \sum_{k=1}^{M}\alpha_{M-k+1}^{\prime }g\left(f\left(k\right)\right)$$

*(a’) for g(x) decreasing, for any increasing function f(x) the following inequality holds:*

(4) $$\sum_{k=1}^{M}\alpha_{k}g\left(f\left(k\right)\right)\leq \sum_{k=1}^{M}\alpha_{k}^{\prime }g\left(f\left(k\right)\right)$$

*(b’) for g(x) decreasing, for any increasing function f(x) the following inequality holds:*

(5) $$\sum_{k=1}^{M}\alpha_{M-k+1}^{\prime }g\left(f\left(k\right)\right)\leq \sum_{k=1}^{M}\alpha_{M-k+1}g\left(f\left(k\right)\right)$$

*(c) the following inequalities hold:*

(6) $$\begin{array}{ccc} \alpha_{1}^{\prime } & \geq & \alpha_{1}\\ \alpha_{1}^{\prime }+\alpha_{2}^{\prime } & \geq & \alpha_{1}+\alpha_{2}\\ & \vdots & \\ \alpha_{1}^{\prime }+\ldots +\alpha_{M-1}^{\prime } & \geq & \alpha_{1}+\ldots +\alpha_{M-1}\\ \alpha_{1}^{\prime }+\ldots +\alpha_{M-1}^{\prime }+\alpha_{M}^{\prime } & = & \alpha_{1}+\ldots +\alpha_{M-1}+\alpha_{M} \end{array}$$

*(d) the following inequalities hold:*

(7) $$\begin{array}{ccc} \alpha_{M}^{\prime } & \leq & \alpha_{M}\\ \alpha_{M}^{\prime }+\alpha_{M-1}^{\prime } & \leq & \alpha_{M}+\alpha_{M-1}\\ \vdots \\ \alpha_{M}^{\prime }+\ldots +\alpha_{2}^{\prime } & \leq & \alpha_{M}+\ldots +\alpha_{2}\\ \alpha_{M}^{\prime }+\alpha_{M-1}^{\prime }+\ldots +\alpha_{1}^{\prime } & = & \alpha_{M}+\alpha_{M-1}+\ldots +\alpha_{1} \end{array}$$

*If f(x) is decreasing, the inequalities in a) through b’) are reversed.*

**Proof.**  Indirect or partial proofs can be found in [17,20]. We provide a complete proof in the Appendix A. □

**Note.**  *Observe that in Lemma 1, it is sufficient to consider the monotonicity of the sequences ${\left\{f\left(k\right)\right\}}_{k=1}^{M}$. Furthermore, Lemma 1 can be extended to any real sequences $\left\{y_{k}\right\}$ and $\left\{x_{k}\right\}$, such that $\sum_{k=1}^{M}y_{k}=\sum_{k=1}^{M}x_{k}$. It is enough to observe that the order of all the inequalities are unchanged by adding a positive constant, so that $\left\{y_{k}\right\}$ and $\left\{x_{k}\right\}$ can be made positive, and also are unchanged by the multiplication of a positive constant, so that the resulting $\left\{y_{k}\right\}$ and $\left\{x_{k}\right\}$ sequences can be normalized.*

**Theorem** **1.** *Given a mean M with strictly monotonic function g(x) and two distributions $\left\{\alpha_{k}^{\prime }\right\},\left\{\alpha_{k}\right\}$, the following propositions are equivalent:*

*(a) for all increasing functions f(x)*

(8) $$M\left(\left\{f\left(k\right)\right\},\left\{\alpha_{k}^{\prime }\right\}\right)\leq M\left(\left\{f\left(k\right)\right\},\left\{\alpha_{k}\right\}\right)$$

*(b) for all increasing functions f(x)*

(9) $$M\left(\left\{f\left(k\right)\right\},\left\{\alpha_{M-k+1}\right\}\right)\leq M\left(\left\{f\left(k\right)\right\},\left\{\alpha_{M-k+1}^{\prime }\right\}\right)$$

*(a’) for all decreasing functions f(x)*

(10) $$M\left(\left\{f\left(k\right)\right\},\left\{\alpha_{k}^{\prime }\right\}\right)\geq M\left(\left\{f\left(k\right)\right\},\left\{\alpha_{k}\right\}\right)$$

*(b’) for all decreasing functions f(x)*

(11) $$M\left(\left\{f\left(k\right)\right\},\left\{\alpha_{M-k+1}\right\}\right)\geq M\left(\left\{f\left(k\right)\right\},\left\{\alpha_{M-k+1}^{\prime }\right\}\right)$$

*(c) Condition (c) of Lemma 1 holds*

**Proof.**  It is a direct consequence of Lemma 1 and the definition of quasi-arithmetic mean, observing that the inverse $g^{-1}\left(x\right)$ of a strictly monotonic increasing (respectively decreasing) function g(x) is also increasing (respectively decreasing). □

## 4. Invariance

**Theorem** **2** (Invariance)**.** *Given two distributions $\left\{\alpha_{k}^{\prime }\right\},\left\{\alpha_{k}\right\}$, and two quasi-arithmetic means M, $M^{🟉}$, the following propositions are equivalent:*

*(a) for all increasing functions f(x)*

(12) $$M\left(\left\{f\left(k\right)\right\},\left\{\alpha_{k}^{\prime }\right\}\right)\leq M\left(\left\{f\left(k\right)\right\},\left\{\alpha_{k}\right\}\right)$$

*(b) for all increasing functions f(x)*

(13) $$M^{🟉}\left(\left\{f\left(k\right)\right\},\left\{\alpha_{k}^{\prime }\right\}\right)\leq M^{🟉}\left(\left\{f\left(k\right)\right\},\left\{\alpha_{k}\right\}\right)$$

*(a’) for all decreasing functions f(x)*

(14) $$M\left(\left\{f\left(k\right)\right\},\left\{\alpha_{k}^{\prime }\right\}\right)\geq M\left(\left\{f\left(k\right)\right\},\left\{\alpha_{k}\right\}\right)$$

*(b’) for all decreasing functions f(x)*

(15) $$M^{🟉}\left(\left\{f\left(k\right)\right\},\left\{\alpha_{k}^{\prime }\right\}\right)\geq M^{🟉}\left(\left\{f\left(k\right)\right\},\left\{\alpha_{k}\right\}\right)$$

**Proof.**  It is a direct consequence of the observation that conditions (c) and (d) in Lemma 1 do not depend on any particular g(x) function considered, and thus the order of inequalities does not change with the mean considered as long as $\left\{\alpha_{k}\right\}$ and $\left\{\alpha_{k}^{\prime }\right\}$ are kept fixed. □

The following properties relate stochastic order with the quasi-arithmetic mean. Let I be the set of monotonous functions, $I^{<}$ the set of increasing functions, $I^{>}$ the set of decreasing functions.

**Definition** **3** (preserve mean order property)**.** *We say that a stochastic order preserves mean order for a given mean M and a set $I^{<}\subset I^{<}$ of increasing functions when, for all functions $f\left(x\right)\in I^{<}$ and any distributions $\left\{\alpha_{k}^{\prime }\right\},\left\{\alpha_{k}\right\}$,*

(16) $$\left\{\alpha_{k}\right\}≺\left\{\alpha_{k}^{\prime }\right\}\Rightarrow M\left(\left\{f\left(k\right)\right\},\left\{\alpha_{k}^{\prime }\right\}\right)\leq M\left(\left\{f\left(k\right)\right\},\left\{\alpha_{k}\right\}\right).$$

**Definition** **4** (preserve inverse mean order property)**.** *We say that a stochastic order preserves inverse mean order for a given mean M and a set $I^{>}\subset I^{>}$ of decreasing functions when, for all functions $f\left(x\right)\in I^{>}$ and any distributions $\left\{\alpha_{k}^{\prime }\right\},\left\{\alpha_{k}\right\}$,*

(17) $$\left\{\alpha_{k}\right\}≺\left\{\alpha_{k}^{\prime }\right\}\Rightarrow M\left(\left\{f\left(k\right)\right\},\left\{\alpha_{k}^{\prime }\right\}\right)\geq M\left(\left\{f\left(k\right)\right\},\left\{\alpha_{k}\right\}\right).$$

Theorem 2 together with the preserve mean order properties allows us to state the following invariance property:

**Theorem** **3** (preserve mean order invariance)**.** *Given a stochastic order that preserves mean order (respectively preserves inverse mean order) for a given mean and for $I^{<}$ (respectively for $I^{>}$), then for any mean it preserves both mean order for $I^{<}$ and inverse mean order for $I^{>}$. In other words, the preserve mean order properties are invariant with respect to the mean considered.*

Observe that from Lemma 1 and Theorems 1 and 3, we have that a necessary and sufficient condition for an order to preserve mean order for $I^{<}$ (or preserve inverse mean order for $I^{>}$) is the holding of Equation (6), independently of the mean considered. We will see in Section 6 that this corresponds to first stochastic dominance order.

## 5. Concavity and Convexity

Let us consider now $I_{v}^{<}\subset I^{<}$, the set of all increasing concave functions, $I_{x}^{<}\subset I^{<}$, the set of all increasing convex functions, $I_{v}^{>}\subset I^{>}$, the set of all decreasing concave functions, and $I_{x}^{>}\subset I^{>}$, the set of all decreasing convex functions. The following theorem relates the preserve mean order properties with the mirror property.

**Theorem** **4.** *If an order holds the mirror property, then holding preserve mean order for $I_{v}^{<}$ (respectively $I_{x}^{<}$) implies holding preserve inverse mean order for $I_{v}^{>}$ (respectively $I_{x}^{>}$) and viceversa.*

**Proof.**  Suppose $\left\{\alpha_{k}^{\prime }\right\}≺\left\{\alpha_{k}\right\}$ and f(x) decreasing and concave (respectively convex). Then, by the mirror property $\left\{\alpha_{M-k+1}\right\}≺\left\{\alpha_{M-k+1}^{\prime }\right\}$, and by the hypothesis of the theorem

(18) $$\begin{array}{ccc} M(\left\{f\left(k\right)\right\},\left\{\alpha_{k}\right\}) & = & M\left(\left\{f\left(M-k+1\right)\right\},\left\{\alpha_{M-k+1}\right\}\right)=M\left(\left\{f\left(m\left(k\right)\right)\right\},\left\{\alpha_{M-k+1}\right\}\right)\\ & \leq & M\left(\left\{f\left(m\left(k\right)\right)\right\},\left\{\alpha_{M-k+1}^{\prime }\right\}\right)=M\left(\left\{f\left(k\right)\right\},\left\{\alpha_{k}^{\prime }\right\}\right), \end{array}$$

where m(x)=M−x+1, and because if f(x) is decreasing and concave (respectively convex) then f(m(x)) is increasing and concave (respectively convex). □

The following result is necessary to prove Lemma 2.

**Theorem** **5.** *Given f(x) and weights $\left\{\alpha_{k}^{\prime }\right\},\left\{\alpha_{k}\right\}$, if inequality $M\left(\left\{f\left(k\right)\right\},\left\{\alpha_{k}\right\}\right)\leq M(\left\{f\left(k\right)\right\},$$\left\{\alpha_{k}\}\right)$ holds for any g(x) strictly increasing and convex (respectively concave) then it holds for any g(x) strictly decreasing and concave (respectively convex). If it holds for any g(x) strictly decreasing and convex (respectively concave) then it holds for any g(x) strictly increasing and concave (respectively convex).*

**Proof.**  Consider the quasi-arithmetic mean with Kolmogorov function $g^{🟉}=-g\left(x\right)$ (with inverse $g^{🟉-1}=g^{-1}\left(-x\right)$). When g(x) is increasing, $g^{🟉}\left(x\right)$ is decreasing and viceversa. When g(x) is convex, $g^{🟉}\left(x\right)$ is concave and viceversa. We have

(19) $$\begin{array}{ccc} g^{🟉-1}\left(\sum_{i}\alpha_{i}^{\prime }g^{🟉}\left(f\left(i\right)\right)\right) & = & g^{-1}\left(-\left(\sum_{i}\alpha_{i}^{\prime }\left(-g\left(f\left(i\right)\right)\right)\right)\right)=g^{-1}\left(\sum_{i}\alpha_{i}^{\prime }g\left(f\left(i\right)\right)\right)\\ & \leq & g^{-1}\left(\sum_{i}\alpha_{i}g\left(f\left(i\right)\right)\right)=g^{-1}\left(-\left(\sum_{i}\alpha_{i}\left(-g\left(f\left(i\right)\right)\right)\right)\right)=g^{🟉-1}\left(\sum_{i}\alpha_{i}g^{🟉}\left(f\left(i\right)\right)\right) \end{array}$$

□

The following Lemma is needed to prove how the invariance properties for one mean extends to other means.

**Lemma** **2.** *Given two distributions $\left\{\alpha_{k}^{\prime }\right\},\left\{\alpha_{k}\right\}$, and a quasi-arithmetic mean $M(\left\{f\left(k\right)\right\},\left\{\alpha_{k}\right\})$ with function g(x). Consider the following Equations:*

(20) $$M\left(\left\{f\left(k\right)\right\},\left\{\alpha_{k}^{\prime }\right\}\right)\leq M\left(\left\{f\left(k\right)\right\},\left\{\alpha_{k}\right\}\right),$$

*and*

(21) $$\begin{array}{c} M\left(\left\{f\left(k\right)\right\},\left\{\alpha_{M-k+1}\right\}\right)\leq M\left(\left\{f\left(k\right)\right\},\left\{\alpha_{M-k+1}^{\prime }\right\}\right). \end{array}$$

*Then, for each line in Table 1, and for g(x),f(x) filling the conditions in first and second column in Table 1 and holding Equations (20) and (21), Equations (20) and (21) hold too for $g^{🟉}\left(x\right),f^{🟉}\left(x\right)$ filling the conditions in columns three and four.*

*Additionally, for each line in Table 1, changing $f^{🟉}\left(x\right)$ in column four from increasing to decreasing, Equations (20) and (21) hold with the inequalities reversed.*

**Proof.**  The proof of lines 1–8 in Table 1 is in the Appendix B. Lines 1’-8’ in Table 1 are a direct consequence of Theorem 5 applied to lines 1–8. □

**Theorem** **6.** *Given a quasi-arithmetic mean $M(\left\{f\left(k\right)\right\},\left\{\alpha_{k}\right\})$ with function g(x), we have that if an order holds the mirror property then for each line in Table 1, and for g(x) filling the condition in the first column in Table 1 and preserving order for f(x) in the second column in Table 1, the mean for $g^{🟉}\left(x\right)$ filling the condition in column three in Table 1 preserves order for $f^{🟉}\left(x\right)$ filling the condition in column four in Table 1.*

**Proof.**  It is enough to apply Lemma 2, taking into account that if Equation (20) holds then Equation (21) holds too by the mirror property. □

**Corollary** **1.** *Consider the weighted arithmetic mean $A(\left\{f\left(k\right)\right\},\left\{\alpha_{k}\right\})$. Given an order that holds the mirror property and preserves the order for mean A, then*

*(a) If order is preserved for mean A and for $I_{v}^{<}$ then order is preserved for any mean with concave-increasing/convex-decreasing (respectively concave-decreasing/convex-increasing) function g(x) and for $I_{v}^{<}$ (respectively for $I_{x}^{<}$).*

*(b) If order is preserved for mean A and for $I_{x}^{<}$, then order is preserved for any mean with convex-increasing/concave-decreasing (respectively convex-decreasing/concave-increasing) function g(x) and for $I_{x}^{<}$ (respectively for $I_{v}^{<}$).*

**Proof.**  Arithmetic mean is a quasi-arithmetic mean with increasing function g(x)=x, which is both concave-increasing and convex-increasing, and thus Table 1 collapses to Table 2.

□

Functions $x^{p}$, for p≥1 are convex-increasing and with p≤0 are convex-decreasing over $R_{++}$, $e^{x}$ convex-increasing over R, while logx, $x^{p}$, for 0<p≤1 are concave-increasing over $R_{++}$. Affine functions are both concave and convex over R. If g(x) is convex, −g(x) is concave and viceversa, and the composition of concave-increasing and concave is concave, and convex-increasing and convex is convex. We will see in the next section that preserving the order for mean A for $I_{v}^{<}$ is defined as second-order stochastic dominance or increasing concave order, and preserving the order for mean A for $I_{x}^{<}$ is defined as increase convex order stochastic dominance. Both orders hold the mirror property and thus Corollary 1 applies to both.

## 6. Application to Stochastic Orders and Cross-Entropy

### 6.1. First-Order Stochastic Dominance

Originating in the economics risk literature [24], first-order stochastic dominance [1,2] FSD, between two probability distributions, $\left\{\alpha_{k}^{\prime }\right\}$, $\left\{\alpha_{k}\right\}$, $\left\{\alpha_{k}^{\prime }\right\}{≺}_{FSD}\left\{\alpha_{k}\right\}$, is defined as:

**Definition** **5.** *$\left\{\alpha_{k}^{\prime }\right\}{≺}_{FSD}\left\{\alpha_{k}\right\}$⇔ for any increasing function f(x),*

$$E{\left[f\left(k\right)\right]}_{\alpha_{k}^{\prime }}\leq E{\left[f\left(k\right)\right]}_{\alpha_{k}}.$$

*Remember that the expected value is the arithmetic weighted mean.*

A necessary and sufficient condition for first-order stochastic dominance is defined by the condition c) of Lemma 1, which is equivalent to condition d) of Lemma 1, thus $\left\{\alpha_{k}^{\prime }\right\}{≺}_{FSD}\left\{\alpha_{k}\right\}\Rightarrow \left\{\alpha_{M-k+1}\right\}{≺}_{FSD}\left\{\alpha_{M-k+1}^{\prime }\right\}$, i.e., the mirror property holds. From the definition of FSD, and from Theorem 3, we can redefine FSD, $\left\{\alpha_{k}^{\prime }\right\}{≺}_{FSD}\left\{\alpha_{k}\right\}$ as there exists a mean M such that, for any increasing function f(x), Equation (16) holds. This is, the definition of FSD is independent of the mean considered, while the original definition relies on the expected value (arithmetic mean). The mean considered can be arithmetic, harmonic, geometric or any other quasi-arithmetic mean.

Let us consider now a strictly monotonous function g(x), and define a generalized cross-entropy $\mathrm{GCE}\left(\left\{p_{i}\right\},\left\{q_{i}\right\}\right)=g^{-1}\left(\sum_{i}p_{i}g\left(-\log q_{i}\right)\right)$. Observe that it is a quasi-arithmetic mean, and for g(x)=x, we get the cross-entropy $CE\left(\left\{p_{i}\right\},\left\{q_{i}\right\}\right)=-\sum p_{i}\log q_{i}$. Other functions that generalize cross entropy have been defined in the context of training deep neural networks [25]. We can state the following theorem:

**Theorem** **7.** *Given distributions $\left\{p_{i}^{\prime }\right\},\left\{p_{i}\right\},\left\{q_{i}\right\}$, increasing, and $\left\{p_{k}^{\prime }\right\}{≺}_{FSD}\left\{p_{k}\right\}$, then for any mean GCE with function g(x), $\mathrm{GCE}\left(\left\{p_{i}\right\},\left\{q_{i}\right\}\right)\leq \mathrm{GCE}\left(\left\{p_{i}^{\prime }\right\},\left\{q_{i}\right\}\right)$.*

**Proof.**  Observe first that $f\left(k\right)=-\log q_{k}$ is a decreasing sequence. From the hypothesis, condition c) of Lemma 1 holds, thus we can then apply Theorem 1 to the mean with function g(x) and for {f(k)} decreasing. □

The following result relates the entropies of two distributions with the first stochastic order.

**Theorem** **8.** *Given $\left\{p_{k}\right\},\left\{q_{k}\right\}$ increasing, and $\left\{q_{k}\right\}{≺}_{FSD}\left\{p_{k}\right\}$, then $H\left(\left\{p_{k}\right\}\right)\leq H\left(\left\{q_{k}\right\}\right)$, where H stands for Shannon entropy.*

**Proof.**  The Kullback–Leibler distance is always positive [26], $\sum_{i}p_{i}\log\frac{p_{i}}{q_{i}}\geq 0$, and thus we have that $H\left(\left\{p_{k}\right\}\right)=-\sum_{i}p_{i}\log p_{i}\leq -\sum p_{i}\log q_{i}$. Applying Theorem 7 for g(x)=x, $-\sum p_{i}\log q_{i}\leq -\sum q_{i}\log q_{i}=H\left(\left\{q_{k}\right\}\right).$ □

### 6.2. Second-Order Stochastic Dominance and Increase Convex Ordering

**Definition** **6.** *Second-order stochastic dominance between two probability distributions, $\left\{\alpha_{k}\right\}$, $\left\{\alpha_{k}^{\prime }\right\}$, $\left\{\alpha_{k}^{\prime }\right\}{≺}_{SSD}\left\{\alpha_{k}\right\}$, occurs when for any increasing concave function f(x),*

$$E{\left[f\left(k\right)\right]}_{\alpha_{k}^{\prime }}\leq E{\left[f\left(k\right)\right]}_{\alpha_{k}}.$$

Trivially

$$\left\{\alpha_{k}^{\prime }\right\}{≺}_{FSD}\left\{\alpha_{k}\right\}\Rightarrow \left\{\alpha_{k}^{\prime }\right\}{≺}_{SSD}\left\{\alpha_{k}\right\}.$$

Let us consider the cumulative distribution function, $F_{i}=\alpha_{1}+\alpha_{2}+\ldots +\alpha_{i}$, and the survival function ${\bar{F}}_{i}=1-F_{i}=\alpha_{M}+\alpha_{M-1}+\ldots +\alpha_{i+1}$. A necessary and sufficient condition for second-order stochastic dominance [1,2] is the following:

(22) $$\begin{array}{ccc} F_{1}^{\prime } & \geq & F_{1}\\ F_{1}^{\prime }+F_{2}^{\prime } & \geq & F_{1}+F_{2}\\ & \vdots & \\ F_{1}^{\prime }+\ldots +F_{M-1}^{\prime } & \geq & F_{1}+\ldots +F_{M-1}\\ F_{1}^{\prime }+\ldots +F_{M-1}^{\prime }+F_{M}^{\prime } & \geq & F_{1}+\ldots +F_{M-1}+F_{M}, \end{array}$$

or equivalently,

(23) $$\begin{array}{ccc} {\bar{F}}_{1}^{\prime } & \leq & {\bar{F}}_{1}\\ {\bar{F}}_{1}^{\prime }+{\bar{F}}_{2}^{\prime } & \leq & {\bar{F}}_{1}+{\bar{F}}_{2}\\ & \vdots & \\ {\bar{F}}_{1}^{\prime }+\ldots +{\bar{F}}_{M-1}^{\prime } & \leq & {\bar{F}}_{1}+\ldots +{\bar{F}}_{M-1}\\ {\bar{F}}_{1}^{\prime }+\ldots +{\bar{F}}_{M-1}^{\prime }+{\bar{F}}_{M}^{\prime } & \leq & {\bar{F}}_{1}+\ldots +{\bar{F}}_{M-1}+{\bar{F}}_{M}. \end{array}$$

Let us see first that $\left\{\alpha_{k}^{\prime }\right\}{≺}_{SSD}\left\{\alpha_{k}\right\}\Rightarrow \left\{\alpha_{M-k+1}\right\}{≺}_{SSD}\left\{\alpha_{M-k+1}^{\prime }\right\}$, i.e., SSD holds the mirror property. Effectively, define $G_{i}=\alpha_{M}+\alpha_{M-1}+\ldots +\alpha_{i+1}$, and the survival function ${\bar{G}}_{i}=1-G_{i}=\alpha_{1}+\alpha_{2}+\ldots +\alpha_{i}$. However, $G_{i}={\bar{F}}_{i}$, ${\bar{G}}_{i}=F_{i}$, $G_{i}^{\prime }={{\bar{F}}^{\prime }}_{i}$, ${{\bar{G}}^{\prime }}_{i}=F_{i}^{\prime }$, and substituting in Equation (22) or Equation (23), we obtain the desired result.

From Corollary 1, second-order stochastic dominance preserves mean order for all means M defined by a concave-increasing/convex-decreasing (respectively concave-decreasing/convex-increasing) function g(x), and for the set of all concave-increasing functions $I_{v}^{<}$ (respectively convex-increasing functions $I_{x}^{<}$). For instance, order is preserved for the geometric mean, or any mean with $g\left(x\right)=x^{p}$, 0<p≤1 and for $I_{v}^{<}$, or for or any mean with $g\left(x\right)=x^{p}$ with p<0, as the harmonic mean, for $I_{x}^{<}$. In particular, we can state the following theorem about cross-entropy of two distributions,

**Theorem** **9.** *Given distributions $\left\{p_{i}^{\prime }\right\},\left\{p_{i}\right\},\left\{q_{i}=f\left(i\right)\right\}$, f(x) concave-increasing, and $\left\{p_{i}^{\prime }\right\}{≺}_{SSD}\left\{p_{i}\right\}$, then $CE\left(\left\{p_{i}\right\},\left\{q_{i}\right\}\right)\leq CE\left(\left\{p_{i}^{\prime }\right\},\left\{q_{i}\right\}\right)$.*

**Proof.**  We have that $CE\left(\left\{p_{i}\right\},\left\{q_{i}\right\}\right)=-\log G\left(\left\{q_{i}\right\},\left\{p_{i}\right\}\right)=-\log\left(\Pi_{i}q_{i}^{p_{i}}\right)=-\log(\exp(\sum_{i}$$p_{i}\log q_{i}))$, where G stands for geometric mean. The geometric mean is a quasi-arithmetic mean with function g(x)=logx, concave-increasing. Using Definition 6 and applying Corollary 1(a), we obtain the inequality $G\left(\left\{q_{i}\right\},\left\{p_{i}^{\prime }\right\}\right)\leq G\left(\left\{q_{i}\right\},\left\{p_{i}\right\}\right)$ and we apply the function −logx to both members of this inequality. □

When we consider f(x), a convex instead of a concave function, we talk of increasing convex order, ICX. This is,

**Definition** **7.** *Second-order stochastic dominance between two probability distributions, $\left\{\alpha_{k}\right\}$, $\left\{\alpha_{k}^{\prime }\right\}$, $\left\{\alpha_{k}^{\prime }\right\}{≺}_{ICX}\left\{\alpha_{k}\right\}$, occurs when for any increasing convex function f(x),*

$$E{\left[f\left(k\right)\right]}_{\alpha_{k}^{\prime }}\leq E{\left[f\left(k\right)\right]}_{\alpha_{k}}.$$

$\left\{\alpha_{k}\right\}$ is greater in increasing convex order than $\left\{\alpha_{k}^{\prime }\right\}$, $\left\{\alpha_{k}^{\prime }\right\}{≺}_{ICX}\left\{\alpha_{k}\right\}$, if and only if the following inequalities hold:

(24) $$\begin{array}{ccc} F_{M}^{\prime } & \geq & F_{M}\\ F_{M}^{\prime }+F_{M-1}^{\prime } & \geq & F_{M}+F_{M-1}\\ & \vdots & \\ F_{M}^{\prime }+\ldots +F_{2}^{\prime } & \geq & F_{M}+\ldots +F_{2}\\ F_{M}^{\prime }+\ldots +F_{2}^{\prime }+F_{1}^{\prime } & \geq & F_{M}+\ldots +F_{2}+F_{1} \end{array}$$

or equivalently,

(25) $$\begin{array}{ccc} {\bar{F}}_{M}^{\prime } & \leq & {\bar{F}}_{M}\\ {\bar{F}}_{M}^{\prime }+{\bar{F}}_{M-1}^{\prime } & \leq & {\bar{F}}_{M}+{\bar{F}}_{M-1}\\ & \vdots & \\ {\bar{F}}_{M}^{\prime }+\ldots +{\bar{F}}_{2}^{\prime } & \leq & {\bar{F}}_{M}+\ldots +{\bar{F}}_{2}\\ {\bar{F}}_{M}^{\prime }+\ldots +{\bar{F}}_{2}^{\prime }+{\bar{F}}_{1}^{\prime } & \leq & {\bar{F}}_{M}+\ldots +{\bar{F}}_{2}+{\bar{F}}_{1} \end{array}$$

Trivially

$$\left\{\alpha_{k}^{\prime }\right\}{≺}_{FSD}\left\{\alpha_{k}\right\}\Rightarrow \left\{\alpha_{k}^{\prime }\right\}{≺}_{ICX}\left\{\alpha_{k}\right\}.$$

Mirror property is also immediate. From Corollary 1, second-order stochastic dominance preserves mean order for all means M defined by a convex-increasing/concave-decreasing (respectively convex-decreasing/concave-increasing) function g(x) and for the set of all convex-increasing functions $I_{x}^{<}$ (respectively concave-increasing functions $I_{v}^{<}$). For instance, any mean with $g\left(x\right)=x^{p}$, p≥1 as the weighted quadratic mean with $I_{x}^{<}$, or $g\left(x\right)=x^{p}$, p<0 as the harmonic mean with $I_{v}^{<}$, or the mean with g(x)=−logx with $I_{v}^{<}$. In particular, we can state the following result,

**Theorem** **10.** *Given distributions $\left\{p_{i}^{\prime }\right\},\left\{p_{i}\right\},\left\{q_{i}=f\left(i\right)\right\}$, f(x) concave-increasing, and $\left\{p_{i}^{\prime }\right\}{≺}_{ICX}\left\{p_{i}\right\}$, then $CE\left(\left\{p_{i}\right\},\left\{q_{i}\right\}\right)\leq CE\left(\left\{p_{i}^{\prime }\right\},\left\{q_{i}\right\}\right)$.*

**Proof.**  Consider the quasi-arithmetic mean $G^{\prime }\left(\left\{q_{i}\right\},\left\{p_{i}\right\}\right)$ with g(x)=−logx, convex-decreasing. We have that $CE\left(\left\{p_{i}\right\},\left\{q_{i}\right\}\right)=-\log G^{\prime }\left(\left\{q_{i}\right\},\left\{p_{i}\right\}\right)$. Using Definition 7 and applying Corollary 1(b), we have that $G^{\prime }\left(\left\{q_{i}\right\},\left\{p_{i}^{\prime }\right\}\right)\leq G^{\prime }\left(\left\{q_{i}\right\},\left\{p_{i}\right\}\right)$, and we apply to each member of the inequality the function −logx. □

### 6.3. Likelihood Ratio Dominance

**Definition** **8.** *Likelihood ratio dominance, LR, $\left\{\alpha_{k}^{\prime }\right\}{≺}_{LR}\left\{\alpha_{k}\right\}$, is defined as*

(26) $$\left\{\alpha_{k}^{\prime }\right\}{≺}_{LR}\left\{\alpha_{k}\right\}\Leftrightarrow \forall \left(i,j\right),1\leq i,j\leq M,i\leq j\Rightarrow \alpha_{i}^{\prime }/\alpha_{j}^{\prime }\geq \alpha_{i}/\alpha_{j}.$$

As $\alpha_{i}^{\prime }/\alpha_{j}^{\prime }\geq \alpha_{i}/\alpha_{j}\Rightarrow \alpha_{j}/\alpha_{i}\geq \alpha_{j}^{\prime }/\alpha_{i}^{\prime }$, we have that LR holds the mirror property.

It can be shown that LR order implies FSD order,

and then LR order holds Theorems 3 and 7 and Equation (16) holds for any mean M,

**Theorem** **11.** *Likelihood-ratio order implies first stochastic order, i.e.,*

(27) $$\left\{\alpha_{k}^{\prime }\right\}{≺}_{LR}\left\{\alpha_{k}\right\}\Rightarrow \left\{\alpha_{k}^{\prime }\right\}{≺}_{FSD}\left\{\alpha_{k}\right\}.$$

**Proof.**  There are indirect proofs in the literature [1,17]. A direct proof is in the Appendix C. □

As the condition for LR order, Equation (26), is easy to check, this order comes very handy in proving sufficient condition for FSD order. Additionally, for the uniform distribution {1/N} and any increasing distribution $\left\{\alpha_{k}\right\}$, we have that $\left\{1/M\right\}{≺}_{LR}\left\{\alpha_{k}\right\}$, while for any decreasing distribution $\left\{\alpha_{k}\right\}$ we have that $\left\{\alpha_{k}\right\}{≺}_{LR}\left\{1/M\right\}$.

Consider Shannon entropy, $H(\left\{p_{k}\right\})$, and Rényi entropy $R_{\beta }\left(\left\{p_{k}\right\}\right)$ of a distribution $\left\{p_{k}\right\}$, that as seen in Section 3 are quasi-arithmetic means of the sequence $\left\{1/\log p_{k}\right\}$ with weights $\left\{p_{k}\right\}$, and with g(x)=x and $g\left(x\right)=2^{(\beta -1)x}$, respectively. Without loss of generality, we can consider $\left\{p_{k}\right\}$ in increasing order, then the sequence $\left\{1/\log p_{k}\right\}$ will be decreasing. We have that $\left\{1/M\right\}≺\left\{p_{k}\right\}$, and then we can apply Theorem 3, and obtain for Shannon entropy

$$H\left(\left\{p_{k}\right\}\right)\leq A\left(\left\{1/\log p_{k}\right\}\right),$$

where $A(\left\{1/\log p_{k}\right\})$ is the arithmetic mean of $\left\{1/\log p_{k}\right\}$, and for Rényi entropy, observing that $g^{-1}\left(x\right)=\frac{1}{1-\beta }\log x$,

$$R_{\beta }\left(\left\{p_{k}\right\}\right)\leq \frac{1}{1-\beta }\log\sum_{k}\frac{1}{M}p_{k}^{\left(\beta -1\right)}=\frac{1}{1-\beta }\log A(\left\{p_{k}^{\left(\beta -1\right)}\right\}.$$

For Tsallis entropy, which can be considered the weighted arithmetic mean of the sequence $\left\{1/\ln_{q}p_{k}\right\}$ with weights $\left\{p_{k}\right\}$, as for $\left\{p_{k}\right\}$ in increasing order the sequence $\left\{1/\ln_{q}p_{k}\right\}$ will be decreasing (the derivative of $\ln_{q}x$ is positive), we have similarly to Shannon entropy,

$$S_{q}\left(\left\{p_{k}\right\}\right)\leq A\left(\left\{1/\ln_{q}p_{k}\right\}\right).$$

We can also state the following result for the generalized cross entropy GCE. We say that $\left\{p_{i}\right\}$ is comonotonic [27,28] with $\left\{q_{i}\right\}$ when, for all 1≤i,j≤M, $q_{i}<q_{j}\Rightarrow p_{i}\leq p_{j}$.

**Theorem** **12.** *Given distributions $\left\{p_{i}\right\},\left\{q_{i}\right\}$ comonotonic, then $\mathrm{GCE}\left(\left\{p_{i}\right\},\left\{q_{i}\right\}\right)\leq \mathrm{GCE}(\left\{1/M\right\},$$\left\{q_{i}\}\right)$.*

**Proof.**  Without loss of generality, we can reorder $\left\{p_{i}\right\},\left\{q_{i}\right\}$ so that both are increasing. We know that $\left\{1/M\right\}{≺}_{LR}\left\{p_{k}\right\}$ and thus $\left\{1/M\right\}{≺}_{FSD}\left\{p_{k}\right\}$. From the definition of ${≺}_{FSD}$, preserve mean order holds for increasing functions and for arithmetic mean, thus we can then apply Theorem 3 for $\left\{-\log q_{i}\right\}$ decreasing. □

From Theorem 12, when $\left\{p_{i}\right\},\left\{q_{i}\right\}$ are comonotonic, then

(28) $$CE\left(\left\{p_{i}\right\},\left\{q_{i}\right\}\right)\leq \frac{1}{M}\sum_{k=1}^{M}-\log q_{k}.$$

### 6.4. Hazard Rate

**Definition** **9.** *A probability distribution $\left\{\alpha_{k}\right\}$ is greater in hazard rate, HR, to $\left\{\alpha_{k}^{\prime }\right\}$, $\left\{\alpha_{k}^{\prime }\right\}{≺}_{HR}\left\{\alpha_{k}\right\}$, if and only if for all j≥i, the following condition is filled*

$$\frac{{{\bar{F}}^{\prime }}_{i}}{{\bar{F}}_{i}}\geq \frac{{{\bar{F}}^{\prime }}_{j}}{{\bar{F}}_{j}}.$$

Observe that this can be written as

$$\frac{{\bar{F}}_{j}}{{{\bar{F}}^{\prime }}_{j}}\geq \frac{{\bar{F}}_{i}}{{{\bar{F}}^{\prime }}_{i}},$$

or

$$\frac{1-F_{j}}{1-{F^{\prime }}_{j}}\geq \frac{1-F_{i}}{1-{F^{\prime }}_{i}},$$

If we consider now the sequences written in inverse order, i.e., $\alpha_{M-i+1},\alpha_{M-i+1}^{\prime }$, it is clear that if i≤j then M−j+1≤M−i+1, and from $1-{\bar{F}}_{j}={\bar{F}}_{M-j+1}$, we get

$$\frac{{\bar{F}}_{M-j+1}}{{{\bar{F}}^{\prime }}_{M-j+1}}\geq \frac{{\bar{F}}_{M-i+1}}{{{\bar{F}}^{\prime }}_{M-i+1}},$$

which means $\left\{\alpha_{M-k+1}\right\}{≺}_{HR}\left\{\alpha_{M-k+1}^{\prime }\right\}$, and HR order holds the mirror property.

It can be shown that $\left\{\alpha_{k}^{\prime }\right\}{≺}_{HR}\left\{\alpha_{k}\right\}\Rightarrow \left\{\alpha_{k}^{\prime }\right\}{≺}_{FSD}\left\{\alpha_{k}\right\}$ and then HR order holds Theorems 3 and 7, and Equation (16) holds for any mean M.

## 7. Example: Linear Combination of Monte Carlo Techniques

When we want to estimate an integral μ=∫h(x)dx using MIS (multiple importance sampling) Monte Carlo methods, we have several choices of techniques, each of them with a given pdf $\left\{p_{k}\left(x\right)\right\}$, 1≤k≤n, which provide the primary estimators $\left\{I_{k,1}=\frac{h(x)}{p_{k}\left(x\right)}\right\}$. If for h(x)>0, we have that $p_{k}\left(x\right)>0$, then the technique is unbiased. We are interested in optimal ways of combining the techniques. One option is linearly combining the different estimators $\left\{I_{i,n_{i}}\right\}$, $\sum_{i=1}^{M}w_{i}I_{i,n_{i}}$, with weights $\left\{w_{k}\right\}$ and sampling proportions $\left\{n_{i}=\alpha_{i}N\right\}$, with $N=\sum_{i=1}^{M}n_{i}$. If all techniques are unbiased, the resulting combination is also unbiased. The variance is given by

(29) $$V_{N}=\sum_{i=1}^{M}w_{i}^{2}\frac{v_{i}}{n_{i}}=\frac{1}{N}\sum_{i=1}^{M}w_{i}^{2}\frac{v_{i}}{\alpha_{i}}=\frac{1}{N}V,$$

where $v_{i}$ are the variances for the primary estimators of each technique and *V* is the variance for the primary MIS estimator.

The optimal combination of weights, i.e. the one that leads to minimum variance, has been studied in [18,29,30].

The variance value will depend on two sets of weights, $\left\{w_{i}\right\}$ and $\left\{\alpha_{i}\right\}$, but there are cases where we can reduce it to a single set of weights. We present the following examples, where variances are taken for N=1:

- when $\alpha_{i}=w_{i}$, then $V=\sum_{i=1}^{M}\alpha_{i}v_{i}$, the weighted arithmetic mean (g(x)=x) of $\left\{v_{i}\right\}$.
- when the sampling proportions are fixed, the optimal variance is given by $H(\left\{\frac{v_{k}}{\alpha_{k}}\right\})$, the weighted harmonic mean (g(x)=1/x) of $\left\{v_{i}\right\}$.
- when weights $\left\{w_{i}\right\}$ are fixed, the optimal variance is given by ${\left(\sum_{k=1}^{M}w_{k}{\sqrt{v}}_{k}\right)}^{2}$, which is the weighted power mean of $\left\{v_{i}\right\}$ with exponent r=1/2 ($g\left(x\right)=x^{1/2}$).

Observe that the variance in these three cases is a quasi-arithmetic mean.

Let us now order $\left\{v_{k}\right\}$ in increasing order, and let us take $\alpha_{k}^{\prime }=h\left(v_{k}\right)$, h(x) decreasing, and $\alpha_{k}=1/M$. We have that $\alpha_{k}^{\prime }=h\left(v_{k}\right){≺}_{LR}\left\{\alpha_{k}=1/M\right\}$ and thus, by Theorem 11, $\alpha_{k}^{\prime }=h\left(v_{k}\right){≺}_{FSD}\left\{\alpha_{k}=1/M\right\}$, and we can apply Theorem 2 with $f\left(k\right)=v_{k}$ to the quasi-arithmetic means above. Thus, the variance is less when taking sampling proportions or coefficients decreasing in $v_{k}$ than for equal sampling or equal weighting.

The same would be the case when considering a different measure of error, whenever the error of the combined techniques can also be expressed as a weighted quasi-arithmetic mean of the values of this measure for all techniques. For instance, suppose we use as measure of error the standard deviation, $\sigma_{i}^{2}=v_{i}$, $\sigma^{2}=V$, the above cases become

- when $\alpha_{i}=w_{i}$, then $\sigma =\sqrt{V}=\sqrt{\sum_{i=1}^{M}\alpha_{i}\sigma_{i}^{2}}$, the weighted root mean square or quadratic mean of $\left\{\sigma_{i}\right\}$, or weighted power mean with r=2 ($g\left(x\right)=x^{2}$).
- when the weights $\left\{w_{i}\right\}$ are fixed, the optimal standard deviation is given by $\sigma =\sqrt{V}=\sqrt{H(\left\{\frac{\sigma_{k}^{2}}{\alpha_{k}}\right\})}$, the power mean of $\left\{\sigma_{i}\right\}$ with r=−2 ($g\left(x\right)=x^{-2}$).
- when the sampling proportions are fixed, the optimal standard deviation is given by $\sigma =\sqrt{V}=\sum_{k=1}^{M}w_{k}\sigma_{k}$, the arithmetic mean of $\left\{\sigma_{i}\right\}$ (g(x)=x).

For more details see [18].

### Liabilities vs. Utilities

From the above example, we can study the relationship between utilities, which we try to maximize, and liabilities, that we try to minimize. As a liability can always be considered as the inverse of a utility, we can see from Theorem 1 that establishing invariance properties on the order between means of utilities is equivalent to doing this with liabilities, except that the order is inverted. We can define for instance the efficiency E=1/V as the inverse of the variance, and obtain it as a weighted mean of individual technique’s efficiencies, $e_{i}=1/v_{i}$. Consider, for instance, the first case in the example above, where $V=\sum_{i=1}^{M}\alpha_{i}v_{i}$. We have

$$E=\frac{1}{V}=\frac{1}{\sum_{i=1}^{M}\alpha_{i}v_{i}}=\frac{1}{\sum_{i=1}^{M}\alpha_{i}e_{i}^{-1}}=H\left(\left\{\frac{e_{i}}{\alpha_{i}}\right\}\right),$$

where H denotes the weighted harmonic mean.

Considering now the second case

$$E=\frac{1}{V}=\frac{1}{H(\left\{\frac{v_{k}}{\alpha_{k}}\right\})}=\sum_{i=1}^{M}\alpha_{i}v_{i}^{-1}=\sum_{i=1}^{M}\alpha_{i}e_{i},$$

or weighted arithmetic mean. For the third case,

$$E=\frac{1}{V}=\frac{1}{{\left(\sum_{k=1}^{M}w_{k}{\sqrt{v}}_{k}\right)}^{2}}={\left(\sum_{k=1}^{M}w_{k}e_{k}^{-1/2}\right)}^{-2},$$

which is the weighted power mean with r=−1/2.

## 8. Conclusions and Future Work

We have presented in this paper the relationship between stochastic orders and quasi-arithmetic means. We have proved several ordering invariance theorems, that show that given two distributions under a certain stochastic order, the ordering of the means is preserved for any quasi-arithmetic mean we might consider, this is, not only for the arithmetic mean (or expected value). We have shown how the results apply to first order, second order, likelihood ratio, hazard-rate, and increasing convex stochastic orders, and its application to cross-entropy. We have also presented an application example based on the linear combination of Monte Carlo estimators, and shown that the invariance allows costs or liabilities to be considered as the symmetric case of utilities.

In the future, we want to generalize our results to spatial weight matrices [31]. The rows in a spatial weight matrix are weights that give the influence of *n* entities over each other. Different weighted means such as arithmetic, harmonic, or geometric [32] can be used to compute this influence. We can thus apply our invariance results to each row. We will also investigate which of our results for Shannon entropy extend to Tsallis entropy too. Both Shannon and Tsallis entropy are weighted arithmetic means [23], and given $\left\{p_{k}\right\}$ monotonic, both $\left\{1/\log p_{k}\right\}$ and $\left\{1/\ln_{q}p_{k}\right\}$ are monotonic too. Finally, we will investigate the invariance of the different stochastic orders under the operations of compositional data [3].

## Figures and Tables

**Table 1:** For each line, for g(x),f(x) filling the conditions in first and second column, then Equations (20) and (21) hold for $g^{🟉},f^{🟉}$ filling the conditions in columns three and four. By changing $f^{🟉}$ from increasing to decreasing, the reverse of Equations (20) and (21) hold. ICX: convex and increasing, ICV: concave and increasing, DCX: convex and decreasing, DCV: concave and decreasing.

	g	f	$\;\;\;\;\;g^{🟉}$	$\;\;\;\;\;f^{🟉}$
1	ICX	ICV	ICV	ICV
1’	"	"	DCX	ICV
2	DCX	ICX	DCV	ICX
2’	"	"	ICX	ICX
3	ICV	ICX	ICX	ICX
3’	"	"	DCV	ICX
4	DCV	ICV	DCX	ICV
4’	"	"	ICV	ICV
5	ICX	ICV	DCV	ICX
5’	"	"	ICX	ICX
6	DCX	ICX	ICV	ICV
6’	"	"	DCX	ICV
7	ICV	ICX	DCX	ICV
7’	"	"	ICV	ICV
8	DCV	ICV	ICX	ICX
8’	"	"	DCV	ICX

**Table 2:** For each line, g(x)=x, f(x) filling the conditions in second column and Equations (20) and (21) holding, then Equations (20) and (21) hold too for $g^{🟉},f^{🟉}$ filling the conditions in columns three and four. ICX: convex and increasing, ICV: concave and increasing, DCX: convex and decreasing, DCV: concave and decreasing.

f	$\;\;\;\;\;g^{🟉}$	$\;\;\;\;\;f^{🟉}$
ICV	ICV	ICV
ICV	DCX	ICV
ICX	ICX	ICX
ICX	DCV	ICX
ICV	DCV	ICX
ICV	ICX	ICX
ICX	DCX	ICV
ICX	ICV	ICV

## Data Availability

Not applicable.

## Appendix A. Proof of Lemma 1

**Proof.** Subtracting 1 from both sides of each inequality proves (c)⇔(d). To prove (a)⇒(c), we proceed in the following way. Consider the increasing sequence {g(f(1)),…,g(f(1)),g(f(M)),…,g(f(M))}, f(1)<f(M) (and thus g(f(1))<g(f(M)) by the strict monotonicity of g(x)), and where g(f(1)) is written *l* times, denote $L=a_{1}+\ldots +a_{l}$, $L^{\prime }=a_{1}^{\prime }+\ldots +a_{l}^{\prime }$. Since $a_{l+1}+\ldots +a_{M}=1-L$, $a_{l+1}^{\prime }+\ldots +a_{M}^{\prime }=1-L^{\prime }$, then (a) gives

$$L^{\prime }g\left(f\left(1\right)\right)+\left(1-L^{\prime }\right)g\left(f\left(M\right)\right)-Lg\left(f\left(1\right)\right)-\left(1-L\right)g\left(f\left(M\right)\right)\leq 0,$$

i.e.,

$$\left(L^{\prime }-L\right)\left(g\left(f\left(1\right)\right)-g\left(f\left(M\right)\right)\right)\leq 0\Rightarrow L^{\prime }\geq L.$$

This proves the first M−1 inequalities in *c*, thus (a)⇒(c). Observe now that

$$L^{\prime }\geq L\Rightarrow 1-L\geq 1-L^{\prime },$$

and thus (a)⇒(d) (although this can be seen directly from (a)⇒(c) and (c)⇔(d). To prove that (b) implies (c) and (d), consider the sequence,

{g(f(1)),…,g(f(1)),g(f(M)),…,g(f(M))},

f(1)<f(M), g(f(M)) is written *l* times, and the same definitions as before for $L,L^{\prime }$, then (b) gives

$$\left(1-L\right)g\left(f\left(1\right)\right)+Lg\left(f\left(M\right)\right)-\left(1-L^{\prime }\right)g\left(f\left(1\right)\right)-L^{\prime }g\left(f\left(M\right)\right)\leq 0,$$

and we proceed as above. Thus, (a)⇒(c). Let us see now that (c) implies (a). Define for 1≤k≤M, $A_{k}=\sum_{j=1}^{k}\alpha_{k},A_{k}^{\prime }=\sum_{j=1}^{k}\alpha_{k}^{\prime }$, and $A_{0}=A_{0}^{\prime }=0$. Then,

(A1) $$\begin{array}{ccc} & & \sum_{k=1}^{M}\alpha_{k}g\left(f\left(k\right)\right)-\sum_{k=1}^{M}\alpha_{k}^{\prime }g\left(f\left(k\right)\right)=\sum_{k=1}^{M}\left(\alpha_{k}-\alpha_{k}^{\prime }\right)g\left(f\left(k\right)\right)\\ & = & \sum_{k=1}^{M}\left(A_{k}-A_{k-1}-{A^{\prime }}_{k}+{A^{\prime }}_{k-1}\right)g\left(f\left(k\right)\right)\\ & = & \sum_{k=1}^{M}\left(A_{k}-{A^{\prime }}_{k}\right)g\left(f\left(k\right)\right)-\sum_{k=1}^{M}\left(A_{k-1}-{A^{\prime }}_{k-1}\right)g\left(f\left(k\right)\right)\\ & = & \sum_{k=1}^{M-1}\left(A_{k}-{A^{\prime }}_{k}\right)g\left(f\left(k\right)\right)-\sum_{k=0}^{M-1}\left(A_{k}-{A^{\prime }}_{k}\right)g\left(f\left(k+1\right)\right)\\ & = & \sum_{k=1}^{M-1}\left(A_{k}-{A^{\prime }}_{k}\right)g\left(f\left(k\right)\right)-\sum_{k=1}^{M-1}\left(A_{k}-{A^{\prime }}_{k}\right)g\left(f\left(k+1\right)\right)\\ & = & \sum_{k=1}^{M-1}\left(A_{k}-{A^{\prime }}_{k}\right)\left(g\left(f\left(k\right)\right)-g\left(f\left(k+1\right)\right)\right)\geq 0, \end{array}$$

as (c) implies that for all *k*, $A_{k}^{\prime }-A_{k}\geq 0$, and {g(f(k))} is an increasing sequence. Thus, (c)⇒(a). Repeating the proof for the sequences $\left\{\alpha_{M-k+1}\right\}$ and $\left\{\alpha_{M-k+1}^{\prime }\right\}$, we obtain that (d)⇒(b). Let g(x) be decreasing. Observe that

$$\sum_{k=1}^{M}\alpha_{M-k+1}g\left(f\left(k\right)\right)=\sum_{k=1}^{M}\alpha_{M-k+1}g\left(f\left(m\left(M-k+1\right)\right)\right)=\sum_{k=1}^{M}\alpha_{k}g\left(f\left(m\left(k\right)\right)\right),$$

where m(k)=M−k+1, and thus $\left(a\right)\Rightarrow \left(b^{\prime }\right)$ because {g(f(m(k)))} is an increasing sequence. Now suppose g(x) is increasing, from

$$\sum_{k=1}^{M}\alpha_{k}g\left(f\left(k\right)\right)=\sum_{k=1}^{M}\alpha_{M-k+1}g\left(f\left(M-k+1\right)\right)=\sum_{k=1}^{M}\alpha_{M-k+1}g\left(f\left(m\left(k\right)\right)\right),$$

we have that $\left(b^{\prime }\right)\Rightarrow \left(a\right)$ because {g(f(m(k)))} is a decreasing sequence. We can show similarly that $\left(b\right)\Leftrightarrow \left(a^{\prime }\right)$. Consider now f(x) decreasing. Reversed (a) is

(A2) $$\sum_{k=1}^{M}\alpha_{k}g\left(f\left(k\right)\right)\leq \sum_{k=1}^{M}\alpha_{k}^{\prime }g\left(f\left(k\right)\right),$$

but it can be written as

(A3) $$\begin{array}{ccc} & & \sum_{k=1}^{M}\alpha_{M-k+1}g\left(f\left(M-k+1\right)\right)\leq \sum_{k=1}^{M}\alpha_{M-k+1}^{\prime }g\left(f\left(M-k+1\right)\right)\\ & = & \sum_{k=1}^{M}\alpha_{M-k+1}g\left(f\left(m\left(k\right)\right)\right)\leq \sum_{k=1}^{M}\alpha_{M-k+1}^{\prime }g\left(f\left(m\left(k\right)\right)\right), \end{array}$$

where {g(f(m(k)))} is an increasing sequence, and thus (c)⇒(a) when f(x) is decreasing and for order of inequality in (a) reversed. The other cases for f(x) decreasing can be analogously proven. □

## Appendix B. Proof of Lines 1–8 from Table 1 in Lemma 2

**Proof.** Let us prove first Lines 1–4 from Table 1. Let g(x) be the strictly monotonic function associated with mean M. Consider the following inequalities. For g(x) increasing, $g^{🟉}$ increasing:

(A4) $$\begin{array}{ccc} \sum_{k=1}^{M}\alpha_{k}^{\prime }g^{🟉}\left(f^{🟉}\left(k\right)\right) & = & \sum_{k=1}^{M}\alpha_{k}^{\prime }g\left(g^{-1}\left(g^{🟉}\left(f^{🟉}\left(k\right)\right)\right)\right)\\ & \leq & \sum_{k=1}^{M}\alpha_{k}g\left(g^{-1}\left(g^{🟉}\left(f^{🟉}\left(k\right)\right)\right)\right)=\sum_{k=1}^{M}\alpha_{k}g^{🟉}\left(f^{🟉}\left(k\right)\right). \end{array}$$

For g(x) decreasing, $g^{🟉}$ decreasing:

(A5) $$\begin{array}{ccc} \sum_{k=1}^{M}\alpha_{k}g^{🟉}\left(f^{🟉}\left(k\right)\right) & = & \sum_{k=1}^{M}\alpha_{k}g\left(g^{-1}\left(g^{🟉}\left(f^{🟉}\left(k\right)\right)\right)\right)\\ & \leq & \sum_{k=1}^{M}\alpha_{k}^{\prime }g\left(g^{-1}\left(g^{🟉}\left(f^{🟉}\left(k\right)\right)\right)\right)\leq \sum_{k=1}^{M}\alpha_{k}^{\prime }g^{🟉}\left(f^{🟉}\left(k\right)\right). \end{array}$$

Observe that the central inequalities in Equations (A4) and (A5) are true whenever Equation (20) holds and viceversa. We are interested in considering all possibilities for it to hold, when $f^{🟉}$ and $f\left(x\right)=g^{-1}\left(h\left(x\right)\right)$ are increasing, with $h\left(x\right)=g^{🟉}\left(f^{🟉}\left(x\right)\right)$. We need first to study the convexity/concavity of the function $g^{-1}\left(h\left(x\right)\right)$. Let us suppose all functions are in $C^{2}$, then $h^{\prime }\left(x\right)={g^{🟉}}^{\prime }\left(f^{🟉}\left(x\right)\right){f^{🟉}}^{\prime }\left(x\right)$, and $h^{\prime \prime }\left(x\right)={g^{🟉}}^{\prime \prime }\left(f^{🟉}\left(x\right)\right){\left({f^{🟉}}^{\prime }\left(x\right)\right)}^{2}+{g^{🟉}}^{\prime }\left(f^{🟉}\left(x\right)\right){f^{🟉}}^{\prime \prime }\left(x\right)$. Dropping the arguments for convenience, we have $h^{\prime \prime }={g^{🟉}}^{\prime \prime }{\left({f^{🟉}}^{\prime }\right)}^{2}+{g^{🟉}}^{\prime }{f^{🟉}}^{\prime \prime }$. For $h^{\prime \prime }$ to be positive (convex) ${g^{🟉}}^{\prime \prime }$ has to be positive (convex) and ${g^{🟉}}^{\prime },{f^{🟉}}^{\prime \prime }$ have to be both positive or negative (thus $g^{🟉}$ convex-increasing, *f* convex, or $g^{🟉}$ convex-decreasing, *f* concave). For $h^{\prime \prime }$ to be negative, ${g^{🟉}}^{\prime \prime }$ has to be negative (concave) and ${g^{🟉}}^{\prime },{f^{🟉}}^{\prime \prime }$ have to have a different sign (thus $g^{🟉}$ concave-increasing, *f* concave, or $g^{🟉}$ concave-decreasing, *f* convex). For $g^{-1}\left(h\left(x\right)\right)$, we can apply the same rule. Observe also that the composition of two increasing or two decreasing functions is increasing, and increasing and decreasing or viceversa is decreasing. For the inverse function, as $(g^{-1}\left(g\left(x\right)\right)=x$ we have that ${g^{-1}}^{\prime }\left(g\left(x\right)\right)g^{\prime }\left(x\right)=1$, ${g^{-1}}^{\prime \prime }\left(g\left(x\right)\right){\left(g^{\prime }\left(x\right)\right)}^{2}+{g^{-1}}^{\prime }\left(g\left(x\right)\right)g^{\prime \prime }\left(x\right)=0$, which isolating ${g^{-1}}^{\prime \prime }\left(g\left(x\right)\right)$ and dropping arguments becomes ${g^{-1}}^{\prime \prime }=-{g^{-1}}^{\prime }g^{\prime \prime }$, and thus for $g^{-1}$ convex-increasing (concave-increasing), the inverse is concave-increasing (convex-increasing), while for $g^{-1}$ convex-decreasing (concave-decreasing), the inverse is convex-decreasing (concave-decreasing). In Table A1, we show the different possibilities for f(x) to be increasing when $f^{🟉}$ is increasing. □

**Table A1:** Different possible combinations where the concavity/convexity of $g^{-1}\left(h\left(x\right)\right)$ can be predicted for g(x) and $f^{🟉},f\left(x\right)$ increasing. ICX: convex and increasing, ICV: concave and increasing, DCX: convex and decreasing, DCV: concave and decreasing.

	*g*	$g^{-1}$	$g^{🟉}$	$f^{🟉}$	$h=g^{🟉}\circ f^{🟉}$	$f=g^{-1}\circ h$
1	ICX	ICV	ICV	ICV	ICV	ICV
2	DCX	DCX	DCV	ICX	DCV	ICX
3	ICV	ICX	ICX	ICX	ICX	ICX
4	DCV	DCV	DCX	ICV	DCX	ICV

Let us prove now Lines 5–8 from Table 1.

Consider the following inequalities, where m(x)=M−x+1, for g(x) increasing, $g^{🟉}$ decreasing,

(A6) $$\begin{array}{ccc} \sum_{k=1}^{M}\alpha_{k}g^{🟉}\left(f^{🟉}\left(k\right)\right) & = & \sum_{k=1}^{M}\alpha_{M-k+1}g^{🟉}\left(f^{🟉}\left(M-k+1\right)\right)\\ & = & \sum_{k=1}^{M}\alpha_{M-k+1}g\left(g^{-1}\left(g^{🟉}\left(f^{🟉}\left(m\left(k\right)\right)\right)\right)\right)\leq \sum_{k=1}^{M}\alpha_{M-k+1}^{\prime }g\left(g^{-1}\left(g^{🟉}\left(f^{🟉}\left(m\left(k\right)\right)\right)\right)\right)\\ & = & \sum_{k=1}^{M}\alpha_{M-k+1}^{\prime }g^{🟉}\left(f^{🟉}\left(M-k+1\right)\right)=\sum_{k=1}^{M}\alpha_{k}^{\prime }g^{🟉}\left(f^{🟉}\left(k\right)\right), \end{array}$$

and for g(x) decreasing, $g^{🟉}$ increasing

(A7) $$\begin{array}{ccc} \sum_{k=1}^{M}\alpha_{k}^{\prime }g^{🟉}\left(f^{🟉}\left(k\right)\right) & = & \sum_{k=1}^{M}\alpha_{M-k+1}^{\prime }g^{🟉}\left(f^{🟉}\left(M-k+1\right)\right)\\ & = & \sum_{k=1}^{M}\alpha_{M-k+1}^{\prime }g\left(g^{-1}\left(g^{🟉}\left(f^{🟉}\left(m\left(k\right)\right)\right)\right)\right)\leq \sum_{k=1}^{M}\alpha_{M-k+1}g\left(g^{-1}\left(g^{🟉}\left(f^{🟉}\left(m\left(k\right)\right)\right)\right)\right)\\ & = & \sum_{k=1}^{M}\alpha_{M-k+1}g^{🟉}\left(f^{🟉}\left(M-k+1\right)\right)=\sum_{k=1}^{M}\alpha_{k}g^{🟉}\left(f^{🟉}\left(k\right)\right). \end{array}$$

Observe that the central inequalities in Equations (A6) and (A7) are true whenever Equation (21) holds and viceversa. We are interested in considering all possibilities for it to hold, when $f^{🟉}$ and $f\left(x\right)=g^{-1}\left(h\left(x\right)\right)$ are increasing, with $h\left(x\right)=g^{🟉}\left(f^{🟉}\left(m\left(x\right)\right)\right)$. As before, we need first to study the convexity/concavity of the function $g^{-1}\left(h\left(x\right)\right)$.

Consider the function $h\left(x\right)=g^{🟉}(f^{🟉}\left(m\left(x\right)\right)$. The second derivative is, as $m^{\prime }\left(x\right)=-1$ and $m^{\prime \prime }\left(x\right)=0$, $h^{\prime \prime }\left(x\right)={g^{🟉}}^{\prime \prime }(f^{🟉}\left(m\left(x\right)\right){f^{🟉}}^{\prime 2}\left(m\left(x\right)\right)+{g^{🟉}}^{\prime }(f^{🟉}\left(m\left(x\right)\right){f^{🟉}}^{\prime \prime }\left(m\left(x\right)\right)$. Let us drop the arguments for convenience, then $h^{\prime \prime }={g^{🟉}}^{\prime \prime }{f^{🟉}}^{\prime 2}+{g^{🟉}}^{\prime }{f^{🟉}}^{\prime \prime }$. Observe that it is the same result obtained above. In Table A2, we see the possible combinations for f(x) to be increasing when $f^{🟉}\left(x\right)$ is increasing. The difference with Table A1 is that we have to consider here m(x).

**Table A2:** Different possible combinations where the concavity/convexity of $g^{-1}\left(h\left(x\right)\right)$ can be predicted and f(x) is increasing when g(x) is increasing and $g^{🟉}\left(x\right)$ decreasing (or viceversa) when $f^{🟉}$ is increasing. ICX: convex and increasing, ICV: concave and increasing, DCX: convex and decreasing, DCV: concave and decreasing.

	*g*	$g^{-1}$	$g^{🟉}$	$f^{🟉}$	$f^{🟉}\left(m\right)$	$h=g^{🟉}\circ f^{🟉}\circ m$	$f=g^{-1}\circ h$
5	ICX	ICV	DCV	ICX	DCX	ICV	ICV
6	DCX	DCX	ICV	ICV	DCV	DCV	ICX
7	ICV	ICX	DCX	ICV	DCV	ICX	ICX
8	DCV	DCV	ICX	ICX	DCX	DCX	ICV

Then, we can summarize the results from Table A1 and Table A2 in Table 1. This will do to prove Equation (20). The results for Equation (21) are obtained as above by substituting $\left\{\alpha_{k}\right\},\left\{\alpha_{k}^{\prime }\right\}$ by $\left\{\alpha_{M-k+1}^{\prime }\right\},\left\{\alpha_{M-k+1}\right\}$.

Suppose now $f^{🟉}\left(x\right)$ is decreasing and that Equations (20) and Equation (21) hold for $g^{🟉}\left(x\right)$ and for $f^{🟉}\left(x\right)$ increasing. Then, the following inequalities, for $g^{🟉}\left(x\right)$ increasing,

(A8) $$\begin{array}{ccc} \sum_{k}\alpha_{k}g^{🟉}\left(f^{🟉}\left(k\right)\right) & = & \sum_{k}\alpha_{M-k+1}g^{🟉}\left(f^{🟉}\left(m\left(k\right)\right)\right)\\ & \leq & \sum_{k}\alpha_{M-k+1}^{\prime }g^{🟉}\left(f^{🟉}\left(m\left(k\right)\right)\right)=\sum_{k}\alpha_{k}^{\prime }g^{🟉}\left(f^{🟉}\left(k\right)\right), \end{array}$$

(A9) $$\begin{array}{ccc} \sum_{k}\alpha_{M-k+1}^{\prime }g^{🟉}\left(f^{🟉}\left(k\right)\right) & = & \sum_{k}\alpha_{k}^{\prime }g^{🟉}\left(f^{🟉}\left(m\left(k\right)\right)\right)\\ & \leq & \sum_{k}\alpha_{k}g^{🟉}\left(f^{🟉}\left(m\left(k\right)\right)\right)=\sum_{k}\alpha_{M-k+1}g^{🟉}\left(f^{🟉}\left(k\right)\right), \end{array}$$

and for $g^{🟉}\left(x\right)$ decreasing,

(A10) $$\begin{array}{ccc} \sum_{k}\alpha_{k}^{\prime }g^{🟉}\left(f^{🟉}\left(k\right)\right) & = & \sum_{k}\alpha_{M-k+1}^{\prime }g^{🟉}\left(f^{🟉}\left(m\left(k\right)\right)\right)\\ & \leq & \sum_{k}\alpha_{M-k+1}g^{🟉}\left(f^{🟉}\left(m\left(k\right)\right)\right)=\sum_{k}\alpha_{k}g^{🟉}\left(f^{🟉}\left(k\right)\right), \end{array}$$

(A11) $$\begin{array}{ccc} \sum_{k}\alpha_{M-k+1}g^{🟉}\left(f^{🟉}\left(k\right)\right) & = & \sum_{k}\alpha_{k}g^{🟉}\left(f^{🟉}\left(m\left(k\right)\right)\right)\\ & \leq & \sum_{k}\alpha_{k}^{\prime }g^{🟉}\left(f^{🟉}\left(m\left(k\right)\right)\right)=\sum_{k}\alpha_{M-k+1}^{\prime }g^{🟉}\left(f^{🟉}\left(k\right)\right), \end{array}$$

hold because $f^{🟉}\left(m\left(x\right)\right)$ is an increasing function. Thus, Equations (20) and (21) hold with the direction of inequality reversed. Observe also that $f^{🟉}\left(m\left(x\right)\right)$ will have the same character of convexity/concavity than $f^{🟉}\left(x\right)$. Applying Equations (A8)–(A11), according to whether $g^{🟉}$ is increasing or decreasing, to each line of Table 1, we obtain the desired result.

## Appendix C. Proof of Theorem 11

**Proof.** First, note that if $\frac{\alpha_{i}^{\prime }}{\alpha_{j}^{\prime }}\geq \frac{\alpha_{i}}{\alpha_{j}}$ for i≤j, then $\alpha_{1}^{\prime }\geq \alpha_{1}$ and $\alpha_{M}^{\prime }\leq \alpha_{M}$, see [17]. Let us proceed now by induction. For M=2, the condition c) of Lemma 1 is true, because $\alpha_{1}^{\prime }\geq \alpha_{1}$, and $\alpha_{1}^{\prime }+\alpha_{2}^{\prime }=\alpha_{1}+\alpha_{2}$. Let us suppose that it is true for M−1. If ${\left\{\alpha_{k}^{\prime }\right\}}_{k=1}^{M}{≺}_{LR}{\left\{\alpha_{k}\right\}}_{k=1}^{M}$, then it is immediate that ${\left\{\alpha_{k}^{\prime }/\sum_{k=1}^{M-1}\alpha_{k}^{\prime }\right\}}_{k=1}^{M-1}{≺}_{LR}{\left\{\alpha_{k}/\sum_{k=1}^{M-1}\alpha_{k}\right\}}_{k=1}^{M-1}$, and by the induction hypothesis, the condition c) of Lemma 1 holds, and thus

(A12) $$\begin{array}{ccc} \alpha_{1}^{\prime }/\sum_{k=1}^{M-1}\alpha_{k}^{\prime } & \geq & \alpha_{1}/\sum_{k=1}^{M-1}\alpha_{k}\\ \left(\alpha_{1}^{\prime }+\alpha_{2}^{\prime }\right)/\sum_{k=1}^{M-1}\alpha_{k}^{\prime } & \geq & \left(\alpha_{1}+\alpha_{2}\right)/\sum_{k=1}^{M-1}\alpha_{k}\\ \ldots & \geq & \ldots \\ \left(\alpha_{1}^{\prime }+\alpha_{2}^{\prime }+\ldots +\alpha_{M-1}^{\prime }\right)/\sum_{k=1}^{M-1}\alpha_{k}^{\prime } & = & \left(\alpha_{1}+\alpha_{2}+\ldots +\alpha_{n-1}\right)/\sum_{k=1}^{M-1}\alpha_{k}. \end{array}$$

However, we know that $\alpha_{M}^{\prime }\leq \alpha_{M}$, and thus $\sum_{k=1}^{M-1}\alpha_{k}^{\prime }\geq \sum_{k=1}^{M-1}\alpha_{k}$ because $\sum_{k=1}^{M-1}\alpha_{k}^{\prime }=\sum_{k=1}^{M-1}\alpha_{k}=1$, and thus we obtain condition c) of Lemma 1. □
